# Clinical and Meibomian Lipidomic Changes After Combined Intense Pulsed Light and Triamcinolone Acetonide Therapy for Chalazion: A Retrospective Single‐Arm Pre–Post Case Series

**DOI:** 10.1155/joph/2855851

**Published:** 2026-07-21

**Authors:** Zhenhe Dong, Linxiao Wu, Cheng Zhang, Ran Feng, Huilan Zhang, Yongmei Sang, Yong Tao, Yan Zhang, Tao Zhang

**Affiliations:** ^1^ Beijing Yangfangdian Hospital, Beijing, China; ^2^ Sports & Medicine Integration Research Center (SMIRC), Capital University of Physical Education and Sports, Beijing 100191, China, cupes.edu.cn; ^3^ Taizhou Vocational College of Science & Technology, Taizhou Zhejiang, 318020, China; ^4^ School of Physical Education, Shandong University of Technology, Zibo Shandong, 255000, China, sdut.edu.cn; ^5^ Beijing Chao-Yang Hospital of Capital Medical University, Beijing, China; ^6^ Emerging Interdisciplinary Platform for Medicine and Engineering in Sports (EIPMES), Beijing, China

**Keywords:** chalazion, IPL, lipidomics, meibomian gland, TA

## Abstract

**Purpose:**

To explore the clinical changes associated with intense pulsed light (IPL) combined with triamcinolone acetonide (TA) injections in patients with chalazion and to evaluate associated changes in Hospital Anxiety and Depression Scale (HADS) scores and meibomian gland lipid profiles.

**Methods:**

This study was designed as a retrospective single‐arm pre–post case series involving 15 adult patients with chalazion who had not received alternative treatments for at least three months. No control group was included. The primary outcome was complete resolution of chalazion at the final follow‐up (T2). Secondary outcomes included changes in ocular surface parameters (OSDI, TBUT, CFS, and SIT), psychological status assessed using the HADS, and alterations in meibomian gland lipid composition evaluated by lipidomic analysis.

**Results:**

All patients met the predefined criteria for complete chalazion resolution at the final follow‐up (T2). Significant improvements were observed, with OSDI scores decreasing from 26.27 ± 5.11 to 16.6 ± 10.42, TBUT increasing from 3.23 ± 1.05 to 7.53 ± 2.75 s, and CFS reducing from 1 ± 1.13 to 0.27 ± 0.7. Depression (HADS‐D) and anxiety (HADS‐A) scores also decreased significantly. The SIT increased from 9.87 ± 5.05 to 11.27 ± 7.32, though not statistically significant. Lipidomics analysis identified 719 lipids, with significant reductions in sphingolipids (Cer and SM) and glycerophospholipids (LPC, LPE, PC, and PE) posttreatment (*p* < 0.05). A negative correlation was found between DG and TBUT, and a positive correlation between MGDG O and CFS.

**Conclusion:**

In this retrospective single‐arm pre–post case series, IPL combined with TA treatment was associated with the resolution of chalazion and improvements in several clinical and lipidomic parameters. However, these findings should be considered exploratory and interpreted with caution, as spontaneous chalazion resolution and concurrent warm compress therapy may have contributed to the observed changes.

## 1. Introduction

A chalazion is an idiopathic, chronic, nonsuppurative inflammatory condition of the meibomian gland [1]. It arises when blockages at the gland orifices prevent secretions from being discharged smoothly, leading to accumulation within the glands and irritation of surrounding tissues. This results in idiopathic, aseptic, and chronic granulomatous inflammation [2]. In Asia, the incidence of chalazion is approximately 4% [3]. Although generally benign, chalazion can pose significant risks to ocular health. Persistent chalazia increase the risk of meibomian gland infections and exacerbate eyelid inflammation. Moreover, the pressure and irritation from chalazion can cause discomfort in the eyelids and may impair normal vision function [4]. Repeated episodes can lead to more severe clinical symptoms and psychological issues, significantly affecting patients’ ocular health and quality of life.

Chalazions are typically treated with local injections of triamcinolone acetonide (TA) and through surgical interventions [5]. Incision and curettage remain the conventional gold‐standard treatment for chalazion, with generally high success rates but potential risks including bleeding, scarring, infection, and recurrence. Recurrence has been reported following both surgical and conservative treatments, highlighting the need for alternative minimally invasive therapeutic approaches. Previous comparative studies have demonstrated that both incision and curettage and intralesional TA injection can achieve high rates of chalazion resolution, whereas conservative management with warm compresses alone is generally associated with lower success rates and a longer treatment course [6, 7]. Nevertheless, each treatment modality has specific advantages and limitations, and an optimal management strategy remains under discussion.

However, surgical treatment alone can be painful and may cause trauma to the eyelid skin. Recurrence has also been reported following surgical treatment. While local injections of TA can temporarily resolve chalazion, they are prone to recurrence if the obstructed meibomian gland is not cleared. Recently, numerous studies have demonstrated the efficacy of intense pulsed light (IPL) therapy in treating dry eye associated with meibomian gland dysfunction (MGD) [8, 9]. Therefore, combined treatment with IPL and TA may not only improve MGD and tear film stability but may also contribute to chalazion resolution without the need for surgical removal. This approach could provide an effective alternative treatment for chalazions.

In this study, we treated patients with chalazions using a combination of IPL and local injections of TA. We conducted comprehensive evaluations of clinical signs, psychological status, and changes in the lipid composition of the meibomian glands after three treatments. The results of this study may provide preliminary insights into treatment‐associated lipid alterations in chalazion and may help guide future mechanistic and therapeutic research.

## 2. Patients and Methods

### 2.1. Patients

**FIGURE 1:**
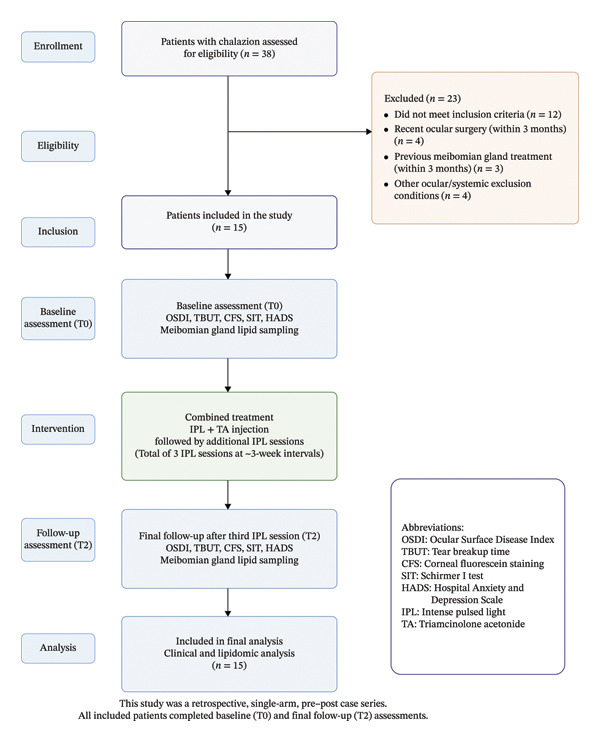
Flow diagram of patient selection and study inclusion.

Patients who received IPL combined with TA treatment for chalazion over the study period at our institution were retrospectively reviewed. This study enrolled adult patients diagnosed with chalazions who had not received any alternative treatments for at least 3 months. The inclusion criteria were as follows: (1) age over 18 years, (2) first diagnosis of a chalazion, (3) duration of the condition for more than 1 month, (4) ability to complete follow‐up examinations, and (5) provision of signed informed consent. The exclusion criteria included the following: (1) abnormalities in eyelid structure or function, (2) eye surgery within the past 3 months, (3) previous meibomian gland treatment, (4) presence of other ocular diseases besides MGD, (5) systemic diseases affecting the eyes, (6) current pregnancy or breastfeeding, and (7) acute inflammation of the eye. The flow diagram of patient selection and inclusion is shown in Figure 1.

After fully explaining the nature of the study and potential consequences, all participants provided signed informed consent. The study was approved by the Institutional Review Board of Beijing Chaoyang Hospital, Capital Medical University (Beijing, China), and adhered to the principles of the Helsinki Declaration. All data were anonymized prior to analysis, and consent included the use of de‐identified clinical data and images where applicable.

### 2.2. Procedures

The IPL treatment protocol includes three sessions per patient, spaced 3 weeks apart. IPL treatment was performed using the M22 IPL system. The procedure is as follows: (1) Instill one drop of procaine hydrochloride eye drops into the conjunctival sac, repeating this twice. (2) Apply a Jaeger eyelid plate coated with ofloxacin eye ointment as ocular protection during IPL treatment. (Suzhou Mingren Medical Equipment Co., Ltd., Suzhou, China). (3) The IPL treatment, performed by the same ophthalmologist, involves (i) cleaning and wiping the patient’s periocular and facial skin; (ii) instructing the patient to relax and keep their eyes closed; (iii) setting the machine parameters to use a three‐pulse mode with an energy density between 14 and 16 J/cm^2^. The filter wavelength, pulse duration, and Fitzpatrick skin type classification were not systematically recorded in the original medical records because of the retrospective nature of the study; and (iv) applying coupling gel to the treatment area, then placing the light guide crystal firmly against the skin surface. Initial testing with parameters below 12 J helps avoid discomfort around the eyebrows, eyelashes, and dense facial hair. If the patient shows no adverse reactions, the full treatment proceeds. The physician places the light guide crystal vertically on the target skin areas of both the upper and lower eyelid margins, covering from the temporal to nasal sides across 12 treatment zones, and repeats these steps. (4) Posttreatment, the coupling gel is removed, and the local skin is disinfected with iodine. A 2–3 mg dose of TA is injected. This suspension should be thoroughly shaken before use. The selected dose was determined based on lesion size and clinical experience. Care is taken with needle direction and depth, inserting from the base of the hard nodule and avoiding any direction toward the eyeball. Postinjection, sterile gauze is applied with gentle pressure for 5–10 min, and patients were advised to apply warm compresses daily for 5–10 min throughout the treatment period. Therefore, warm compress therapy should be considered part of the overall treatment regimen in this study.

### 2.3. Clinical Assessment

Clinical assessments were conducted at two predefined time points: baseline (T0, prior to the first treatment session) and the final follow‐up (T2, after the third IPL session). Ocular surface parameters, including tear breakup time (TBUT), corneal fluorescein staining (CFS) [10], and the Schirmer I test (SIT), as well as subjective symptoms assessed by the Ocular Surface Disease Index (OSDI) questionnaire [11] and psychological status evaluated using the Hospital Anxiety and Depression Scale (HADS) [12], were measured at both T0 and T2. Meibomian gland lipid samples were collected at the same time points for lipidomic analysis. Intermediate posttreatment assessments (e.g., after the first IPL session) were not included in the statistical analysis.

All patients provided their medical history and personal information (including age, gender, surgical history, and systemic and ocular medication history) to confirm eligibility based on the inclusion criteria. Slit‐lamp microscopy was also performed at each visit. Complete resolution of chalazion was defined as the absence of a palpable eyelid nodule and no visible swelling or erythema on slit‐lamp examination at the final follow‐up (T2). Complete resolution was assessed by clinical examination at T2. Because of the retrospective nature of the study, outcome assessors were not masked to treatment timing or baseline clinical findings.

### 2.4. The Collection of Meibomian Gland Lipids

The collection of meibomian gland lipids should be conducted under the following protocol: Patients are prohibited from using cosmetics and artificial tears, and the eyelid margin should be cleaned prior to the collection of lipids. Meibomian gland lipids are collected after the combined IPL and TA treatment. It is important during sample collection and experimentation to avoid using plastic products to prevent lipid contamination. The procedure for collecting lipids is as follows: Use a medical cotton swab to gently scrape the meibomian gland secretions, place the swab in a cryogenic tube, and immediately store it at −80°C for further analysis. All samples were collected using the same standardized procedure by the same investigator. To minimize procedural variability, all samples were collected by the same investigator using a standardized collection protocol. However, the exact pressure applied during meibum expression, the specific gland locations sampled, and the quantity of material collected were not quantitatively standardized or recorded. In addition, lipid sampling was performed from the eyelid margin as a whole and was not restricted to glands corresponding to the chalazion site.

### 2.5. Experimental Methods and Conditions

#### 2.5.1. TripleTOF6600

Lipidomic analysis was performed to explore potential alterations in meibomian gland lipid composition associated with clinical improvement following treatment. We utilized a high‐resolution tandem mass spectrometer, TripleTOF6600 (SCIEX, Framingham, MA, USA), to detect metabolites eluted from the column. The Q‐TOF instrument was operated in both positive and negative ion modes. The settings were as follows: curtain gas at 30 PSI, Ion source gas 1 and Ion source gas 2 both at 60 PSI, and interface heater temperature at 650°C. In positive ion mode, the IonSpray voltage floating was set at 5000 V, and for negative ion mode, it was set at −4500 V. Mass spectrometry data were acquired in IDA mode, with the TOF mass range spanning from 60 to 1200 Da. Survey scans were completed in 150 ms, and up to 12 product ion scans were collected if they exceeded a threshold of 100 counts per second and had a 1+ charge state. The total cycle time was set to 0.56 s. Four time bins were summed for each scan at a pulser frequency value of 11 kHz, monitored through the 40 GHz multichannel TDC detector with four‐anode/channel detection. Dynamic exclusion was set for 4 s. During acquisition, mass accuracy was calibrated every 20 samples. To evaluate the stability of the LC‐MS throughout the acquisition, a quality control sample (a pool of all samples) was analyzed after every 10 samples.

#### 2.5.2. Q‐Exactive

A high‐resolution tandem mass spectrometer, Q‐Exactive (Thermo Scientific), was also used for detecting metabolites eluted from the column. The Q‐Exactive operated in both positive and negative ion modes. Precursor spectra (70–1050 m/z) were collected at a resolution of 70,000 to meet an AGC target of 3e6. The maximum injection time was set at 100 ms. Data acquisition was configured in a top 3 DDA mode. Fragment spectra were collected at a resolution of 17,500 to meet an AGC target of 1e5 with a maximum injection time of 80 ms. Similar to the TripleTOF6600, to assess the LC‐MS stability throughout the entire acquisition, a quality control sample (a pool of all samples) was analyzed after every 10 samples.

### 2.6. Statistical Analysis

Statistical analysis was performed using SPSS Version 22.0 (IBM Corp., Armonk, NY, USA). Continuous variables are presented as mean ± standard deviation. Comparisons of clinical parameters before and after treatment (T0 vs. T2) were performed using paired *t*‐tests for normally distributed data or Wilcoxon signed‐rank tests for non‐normally distributed data. Correlations between lipid species and clinical parameters were assessed using Pearson correlation coefficients for normally distributed variables and Spearman correlation coefficients for non‐normally distributed variables. A *p* value of < 0.05 was considered statistically significant. Due to the exploratory nature and limited sample size of this pilot retrospective study, adjustments for multiple comparisons were not applied to clinical outcome measures. Given the exploratory nature and limited sample size of this pilot study, differential lipid analyses were evaluated using unadjusted *p* values. Therefore, the lipidomic findings should be considered exploratory and interpreted with caution. Correlation analyses between lipid species and clinical parameters were considered exploratory and hypothesis‐generating. Given the limited sample size and multiple comparisons, these findings should be interpreted with caution. No missing data were identified for the primary or secondary outcome measures, and all enrolled patients completed assessments at both T0 and T2. Because this was a retrospective case series, no formal sample size calculation was performed. All eligible patients with complete clinical and lipidomic data during the study period were included in the analysis. This manuscript was prepared in accordance with the STROBE statement for observational studies.

### 2.7. Lipidomic Data Processing

Raw data obtained from LC‐MS/MS were processed using lipid identification software and matched against established lipid databases. Peak detection, alignment, and integration were performed to generate quantitative lipid profiles. Data were normalized using total ion current normalization to reduce intersample variability.

Lipid species with missing values in more than 50% of samples were excluded, and the remaining missing values were imputed using a minimal value approach. Quality control samples were used to monitor system stability throughout the analysis.

Differential lipid analysis between T0 and T2 was performed using appropriate statistical tests. Given the exploratory nature of this pilot study and the limited sample size, results were interpreted primarily based on unadjusted *p* values and should be considered hypothesis‐generating.

## 3. Results

### 3.1. Patient Demographics

At the final follow‐up (T2), complete resolution of chalazion was achieved in all 15 patients based on the predefined clinical criteria. A total of 15 patients were included, with a mean age of 33.47 ± 6.85 years. Meibomian gland lipid samples were collected at baseline (T0) before the first treatment and at the final follow‐up (T2) after the third treatment (Table 1). All patients completed the treatment and evaluation protocol without reporting pain or significant discomfort during the procedures. No treatment‐related adverse events were identified during retrospective review of the available medical records during the available follow‐up period, including skin burns, eyelid depigmentation, infection, ptosis, fat atrophy, or recurrence. Lipidomic analysis was performed using LC‐MS/MS.

**TABLE 1:** Clinical parameters of chalazion patients before and after IPL combined with TA treatment (*n* = 15).

Clinical assessment	Before treatment	After treatment	*T* value	*p* value
Age	33.47 ± 6.85	—	—	—
Gender (male/female)	3/12	—	—	—
OSDI	26.27 ± 5.11	16.6 ± 10.42	3.225	0.009^∗^
TBUT	3.23 ± 1.05	7.53 ± 2.75	−5.661	< 0.001^∗∗∗^
SIT	9.87 ± 5.05	11.27 ± 7.32	−0.609	0.547
CFS	1 ± 1.13	0.27 ± 0.7	2.128	0.042^∗^

*Note:* Data are presented as mean ± standard deviation. OSDI, Ocular Surface Disease Index; TBUT, tear breakup time; SIT, Schirmer’s I test (baseline tear secretion); CFS, corneal fluorescein staining. Mean differences and 95% confidence intervals are provided in the Results section.

^∗^ *p* < 0.05.

^∗∗∗^ *p* < 0.001.

### 3.2. Tear Film and Lipid Analysis

**FIGURE 2:**
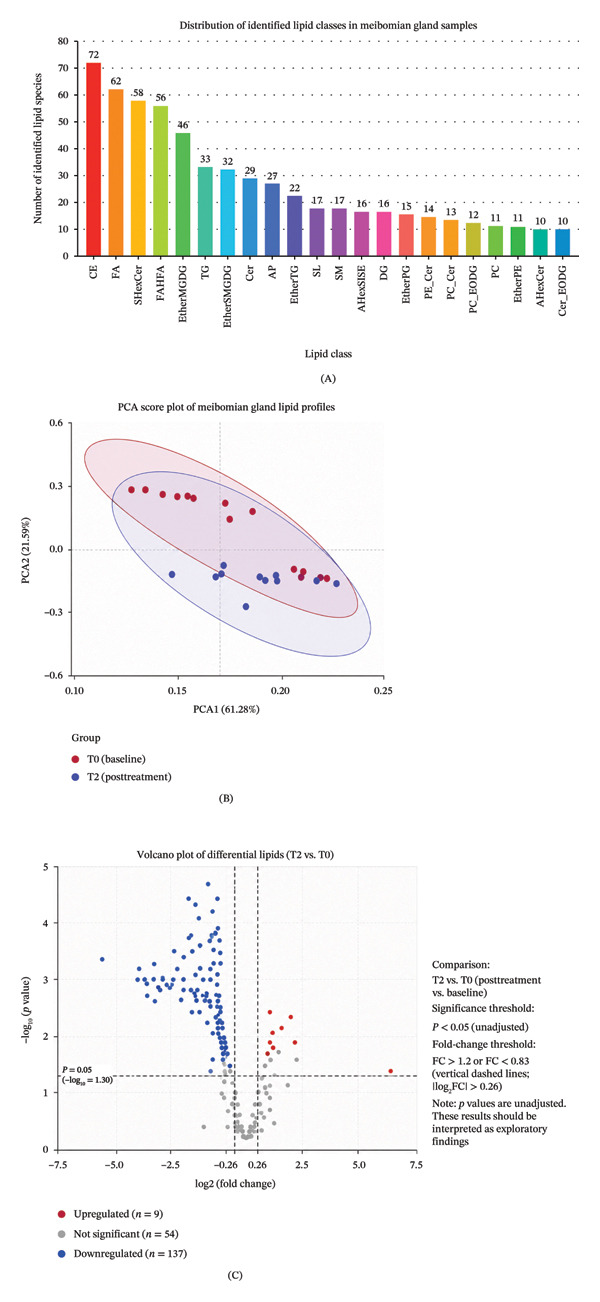
(A) LC‐MS/MS analysis of meibomian gland lipids before the first treatment (T0, *n* = 15) and after the third IPL treatment (T2, *n* = 15). (B) Principal component analysis (PCA) of meibomian gland lipid profiles. T0 represents baseline samples collected before treatment, and T2 represents samples collected after completion of the third IPL session. Substantial overlap between groups was observed, indicating only modest differences in overall lipidomic profiles. (C) Volcano plot showing differential lipid species between baseline (T0) and posttreatment (T2) samples. The *x*‐axis represents log2 fold change, and the *y*‐axis represents −log10 (*p* value). Red points indicate significantly upregulated lipids, blue points indicate significantly downregulated lipids, and gray points indicate lipids that did not meet the predefined significance criteria. Differential lipids were identified using unadjusted *p* values and should be interpreted as exploratory findings.

A total of 719 lipid species were identified, predominantly including triglycerides (TGs), sphingomyelin (SM), ceramide (Cer), phosphatidylcholine (PC), monogalactosyldiacylglycerol (MGDG), lysophosphatidylcholine (LPC), lysophosphatidylethanolamine (LPE), and diglycerides (DGs), among others (Figure 2A). Principal component analysis (PCA) suggested modest differences in lipid profiles between T0 and T2; however, substantial overlap was observed between groups, and the findings should be interpreted cautiously given the limited sample size (Figure 2B). The volcano plot suggested differential lipid expression patterns between T0 and T2; however, these findings should be interpreted cautiously, given the limited sample size (Figure 2C).

**FIGURE 3:**
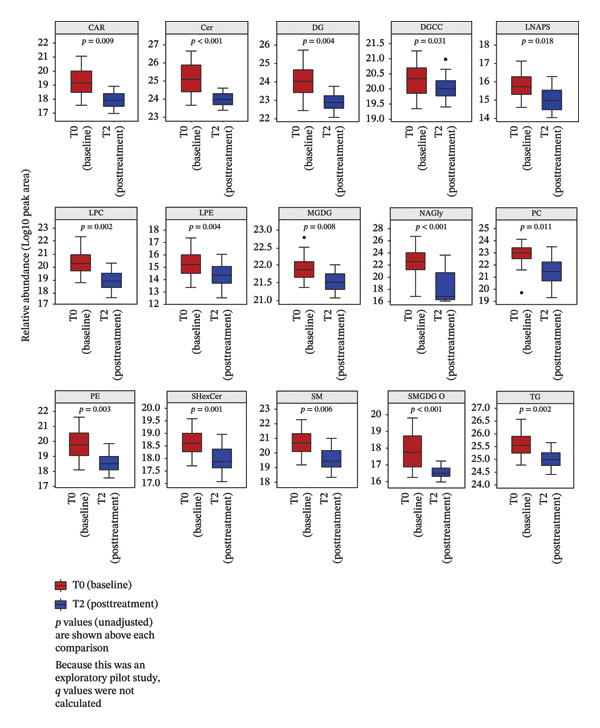
Differential lipid classes between baseline (T0) and posttreatment (T2) samples. Boxplots display the relative abundance of selected lipid classes. Red boxes represent baseline samples (T0), and blue boxes represent posttreatment samples (T2). Statistical comparisons were performed using unadjusted *p* values, which are displayed above each comparison. Because of the exploratory nature of this pilot study, *q* values were not calculated.

At T2, 24 lipid species were significantly altered compared to T0, including TG, LPC, N‐acyl amino acids (OAHFA), Cer, SM, and phosphatidylethanolamines (PEs), among others (Table 2). At the individual lipid level, several lipid classes—including TG, SMGDG O, SM, ShexCer, PE, PC, NAGly, MGDG, LPC, LNAPS, DGCC, DG, Cer, and CAR—were significantly decreased after treatment (Figure 3).

**TABLE 2:** Differential lipid species between baseline (T0) and posttreatment (T2) samples identified using unadjusted *p* values.

LipidIon	Class	Formula	RT	FC	*p* value	VIP
LPE O‐18:1	EtherLPE	C23H48NO6P	3.62	0.50	0.01	1.34
LPC 18:0‐SN1	LPC	C26H54NO7P	3.16	0.22	∼0.001	2.45
SM 8:0; 2O/26:1	SM	C39H79N2O6P	5.94	0.49	0.01	1.44
LPC 16:0‐SN1	LPC	C24H50NO7P	1.87	0.21	∼0.001	2.62
LPC 18:2‐SN1	LPC	C26H50NO7P	2.16	0.40	0.01	1.95
PC 16:0_18:2	PC	C42H80NO8P	5.99	0.52	0.03	1.57
LPE 16:0	LPE	C21H44NO7P	2.58	0.50	0.04	1.23
DG 18:1_18:2	DG	C39H70O5	6.73	0.15	∼0.001	4.71
SHexCer47:2; 3O	SHexCer	C53H101NO12S	2.47	0.25	0.01	2.57
DG 16:0_18:1	DG	C37H70O5	7.01	0.16	∼0.001	3.47
SHexCer45:2; 3O	SHexCer	C51H97NO12S	2.03	0.25	∼0.001	2.02
LPE 18:1	LPE	C23H46NO7P	2.67	0.48	0.03	1.28
PC 34:2	PC	C42H80NO8P	5.99	0.51	0.02	1.70
DG 16:0_18:2	DG	C37H68O5	6.71	0.18	∼0.001	3.39
MGDGO‐8:0_17:2	EtherMGDG	C34H62O9	3.56	0.04	0.01	3.32
PC O‐9:0_9:0	EtherPC	C26H54NO7P	3.17	0.30	∼0.001	2.15
DG 18:1_18:1	DG	C39H72O5	7.02	0.17	∼0.001	3.66
SM 8:0; 2O/26:0	SM	C39H81N2O6P	6.08	0.57	0.02	1.08
MGDGO‐8:0_15:1	EtherMGDG	C32H60O9	3.50	0.03	∼0.001	3.94
TGO‐18:2_8:0_8:0	EtherTG	C37H68O5	6.71	0.35	∼0.001	2.03
TGO‐8:0_16:4_16:4	EtherTG	C43H68O5	2.14	0.20	∼0.001	2.46
PC O‐14:0_2:0	EtherPC	C24H50NO7P	2.48	0.30	0.01	2.22
TGO16:3_10:0_10:0	EtherTG	C39H70O5	6.73	0.34	0.01	2.11

*Note:* There were significant differences in the 24 lipid species between T2 (posttreatment lipids) and T0 (pretreatment lipids), including TG, LPC, OAHFA, Cer, SM, and PE.

**FIGURE 4:**
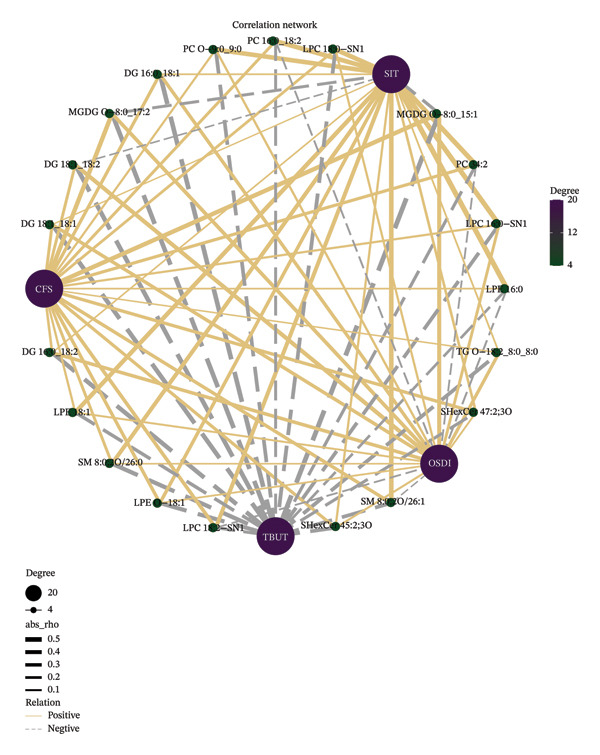
Correlation of important lipid subclasses with clinical indicators. Changes in lipid profiles were associated with changes in clinical indicators. TBUT, SIT, and metabolic quality show rich correlations, with some lipids exhibiting cross‐correlation with them. Purple indicates clinical indicator data, while green represents differential metabolic lipids. The darker the line, the stronger the correlation, with the number on the line indicating the correlation coefficient (*r* value). Solid lines denote positive correlations, while dashed lines denote negative correlations.

**FIGURE 5:**
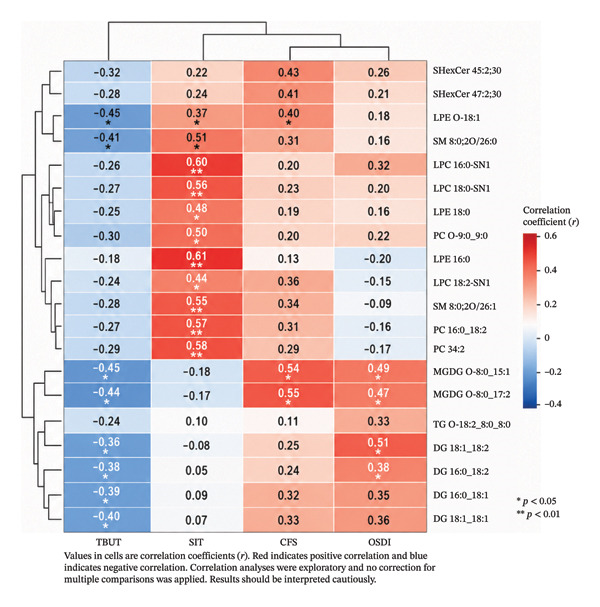
Exploratory correlations between selected lipid species and clinical parameters. The heatmap displays Pearson or Spearman correlation coefficients between lipid species and clinical outcomes (TBUT, SIT, CFS, and OSDI). Red indicates positive correlations and blue indicates negative correlations. Correlation coefficients are shown using the color scale. Statistical significance is indicated as ^∗^ *p* < 0.05 and ^∗∗^ *p* < 0.01. Correlation analyses were exploratory, and no correction for multiple comparisons was applied. Therefore, the observed associations should be interpreted cautiously and considered hypothesis‐generating.

Exploratory correlation analyses suggested potential associations between selected lipid species and clinical parameters; however, these findings should be interpreted cautiously given the limited sample size and the large number of comparisons performed (Figures 4 and 5; Table 3). In particular, TBUT, OSDI, and SIT showed notable correlations with multiple lipid species, with some lipids demonstrating cross‐correlations across these clinical indicators.

**TABLE 3:** Correlation analysis between lipid composition and ophthalmic indicators.

LipidIon	Ophthalmic index	Correlation coefficient	*p* value	Positive/negative correlation
LPE 16:0	SIT	0.57	0.002	Positive
LPC 16:0‐SN1	SIT	0.55	0.003	Positive
PC 34:2	SIT	0.53	0.005	Positive
MGDG O‐8:0_15:1	TBUT	−0.51	0.006	Negative
SM 8:0; 2O/26:1	SIT	0.51	0.008	Positive
LPC 18:0‐SN1	SIT	0.49	0.009	Positive
PC 16:0_18:2	SIT	0.49	0.009	Positive
PC O‐9:0_9:0	SIT	0.49	0.010	Positive
DG 16:0_18:1	TBUT	−0.49	0.010	Negative
MGDG O‐8:0_17:2	TBUT	−0.49	0.010	Negative
DG 18:1_18:2	TBUT	−0.48	0.013	Negative
DG 18:1_18:1	TBUT	−0.47	0.013	Negative
MGDG O‐8:0_17:2	CFS	0.46	0.015	Positive
MGDG O‐8:0_15:1	CFS	0.46	0.010	Positive
DG 16:0_18:2	TBUT	−0.46	0.017	Negative
LPE 18:1	SIT	0.46	0.017	Positive
SM 8:0; 2O/26:0	TBUT	−0.43	0.025	Negative
MGDG O‐8:0_15:1	OSDI	0.43	0.028	Positive
DG 18:1_18:2	OSDI	0.42	0.029	Positive
LPE O‐18:1	TBUT	−0.42	0.030	Negative
SM 8:0; 2O/26:0	SIT	0.42	0.032	Positive
LPC 18:2‐SN1	SIT	0.41	0.034	Positive

*Note:* The correlation between meibomian gland lipid composition and ophthalmic clinical indicators varies significantly from high to low. *p* values shown are unadjusted and should be interpreted as exploratory. Correlation coefficients are reported to two decimal places and *p* values to three decimal places because of the limited sample size.

**FIGURE 6:**
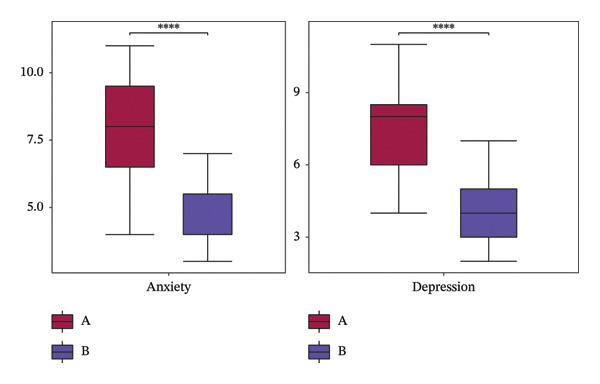
Analysis of patient’s psychological condition. Anxiety and depression scale scores significantly decreased after treatment. A (before treatment) and B (after treatment).

In addition, psychological status improved significantly following treatment. The depression score (HADS‐D) decreased from 7.6 ± 1.8 to 4.13 ± 1.4, and the anxiety score (HADS‐A) decreased from 8.0 ± 2.0 to 4.6 ± 1.2 (both *p* < 0.001), suggesting an improvement in psychological status following treatment (Figure 6).

## 4. Discussion

In this retrospective single‐arm pre–post case series, IPL combined with TA treatment was associated with the resolution of chalazion and improvements in several clinical and lipidomic parameters. Compared to surgical excision, this method may reduce the risk of trauma, infection, and postoperative scarring. In the present study, significant improvements were observed in tear film stability and CFS following treatment, while only a nonsignificant increase in basal tear secretion was observed. In addition, lipidomic analysis revealed significant alterations in several lipid species, including LPC, SM, TG, and Cer, and some of these changes were associated with improvements in clinical parameters. Although several lipid species differed between T0 and T2, the PCA results demonstrated substantial overlap between groups, suggesting that the observed lipidomic changes should not be interpreted as evidence of biologically distinct lipidomic states.

Previous randomized and comparative studies have shown that both incision and curettage and intralesional TA injection are effective treatments for chalazion, with generally comparable resolution rates in many cases. Conservative approaches such as warm compress therapy may be beneficial for selected patients but are often associated with slower or less complete resolution. These findings support continued exploration of minimally invasive treatment strategies that may address both inflammation and MGD [6, 7]

Recent studies have validated the effectiveness of IPL in treating MGD [8, 9]. In addition to studies of MGD and dry eye disease, emerging evidence has suggested a potential role for IPL‐based therapies in the management of chalazion. Song et al. reported that optimal pulse technology, an IPL‐based treatment modality, was associated with favorable clinical outcomes in patients with chalazion and may contribute to lesion resolution through improvement of meibomian gland function and reduction of local inflammation. Although the available evidence remains limited, these findings provide preliminary support for the application of IPL‐related therapies in chalazion management and are consistent with the treatment‐associated improvements observed in the present study [2]. The mechanisms of action include the following: (1) IPL raises the local temperature of the meibomian glands, melting the thickened meibum and facilitating its discharge [13]. (2) The targeted wavelength of IPL disrupts bacterial cell walls, offering antibacterial and anti‐inflammatory effects [14]. (3) Hemoglobin absorbs the yellow light waves passing through the skin, generating heat, which leads to endothelial cell swelling, vascular spasm, and tissue hypoxia. This results in coagulative necrosis and the regression of abnormal new blood vessels on the eyelid margin, reducing inflammatory factors [15]. TA, a synthetic long‐acting glucocorticoid, possesses potent and sustained anti‐inflammatory and antiallergic effects. Local injections of TA effectively inhibit fibroblast proliferation in scars, promote collagen degradation, reduce inflammatory responses, stabilize mast cells, and decrease histamine release [16]. It aids in reducing vascular tension and capillary permeability and inhibits fibroblast production, proving effective in treating chronic inflammation and scar absorption [17]. Research has shown that subcutaneous injections of triamcinolone around a chalazion are more effective than conservative treatments [6]. We hypothesize that IPL may improve MGD, promoting the smooth discharge of meibomian gland lipids and reducing the chronic inflammatory stimulation caused by lipid accumulation, which may contribute to the resolution of chalazion. Concurrently, TA’s anti‐inflammatory and antifibrotic effects may also contribute to chalazion resolution. Although several mechanisms have been proposed based on studies of MGD, ocular surface disease, and dermatologic applications of IPL, direct mechanistic evidence in chalazion remains limited and requires further investigation. This study observed that IPL combined with TA treatment was associated with clinical improvement, including resolution of chalazion, a nonsignificant increase in basal tear secretion, longer tear film breakup time, reduced OSDI scores, decreased corneal staining, and lower HADS scores. However, because of the absence of a control group, the observed improvements in psychological status cannot be directly attributed to the treatment itself and may have been influenced by lesion resolution, expectation effects, regression to the mean, or repeated questionnaire exposure. Previous studies have demonstrated an association between ocular surface symptoms and psychological burden, including anxiety and depression [18, 19]. Therefore, the reduction in HADS scores observed in this study may reflect improvements in ocular symptoms and disease burden rather than a direct psychological effect of the intervention itself.

This study identified a significant decrease in TGs, Cers, and SM in the meibomian gland lipids following treatment. However, because this study did not include healthy controls, untreated chalazion controls, or alternative treatment control groups, it is not possible to determine whether the observed lipid alterations were treatment‐specific, related to chalazion resolution itself, or influenced by sampling variability. TGs, compounds formed by glycerol and three fatty acid molecules connected by ester bonds, represent the main form of fat and serve as a primary energy source in the body [20]. Obstruction or dysfunction of lipid secretion in the meibomian glands of patients with chalazion can lead to lipid accumulation in the gland ducts, ultimately forming cysts [7]. Given that the melting point of TGs is 64.2°C [21], they remain viscous at body temperature and do not easily liquefy, thus accumulating within the meibomian glands. The photothermal effects of IPL have been reported to soften obstructed meibomian gland secretions and facilitate their expression in patients with MGD. Whether these mechanisms directly contribute to chalazion resolution remains to be fully established. The observed reduction in TG content after treatment was temporally associated with clinical improvement; however, the causal relationship between these findings cannot be established in the present study. The study also noted a significant decrease in sphingolipids and glycerophospholipids in the meibomian gland lipids after treatment. Both lipid classes are involved in the structure of biological membranes and are regulated by T cells [22]. Previous research has shown that glycerophospholipids were significantly elevated in meibomian gland lipids of patients with chronic ocular graft‐versus‐host disease (coGVHD) compared to controls, likely due to apoptosis and breakdown of cell membranes in these glands [23]. Similarly, severe meibomian gland apoptosis is observed in chalazion patients. Posttreatment reductions in sphingolipids such as LPC, PC, and PE were observed in the present study. These findings may be consistent with previous reports describing alterations in lipid metabolism and cellular membrane turnover in meibomian gland disorders. However, the clinical significance of these lipid changes and their relationship to chalazion resolution remain uncertain and require further investigation. Cers and SM belong to the class of sphingolipids. Sphingolipids play important roles in various biological processes, including regulating cell apoptosis, growth, inflammatory responses, angiogenesis, and intracellular transport [24–27]. SM is a precursor molecule of Cers [28], and studies have shown that Cers have various proinflammatory and proapoptotic effects [29]. In vitro simulations analyzing the composition of meibomian gland lipid membranes have demonstrated that the artificial addition of Cers significantly disrupts membrane stability, resulting in tear film instability, characterized by reduced tear volume and shortened tear film breakup time [30]. In this study, the observed decrease in Cers and SM content in meibomian gland lipids after treatment, along with improvements in clinical indicators such as basal tear secretion and tear film breakup time, suggests a potential association with improved clinical outcomes. Because the correlation analyses were exploratory and were not adjusted for multiple comparisons, the observed associations should be considered preliminary and require validation in larger independent cohorts. However, these proposed mechanisms cannot be confirmed in the present study and should be interpreted with caution.

This study has several limitations. First, it was a retrospective single‐arm pre–post study with a relatively small sample size, which may limit the generalizability of the findings. Second, the absence of a control group substantially limits the ability to determine whether the observed improvements were attributable to the intervention itself or to the natural resolution of chalazion. The observed improvements may have been influenced by potential confounding factors, including regression to the mean, the natural course of chalazion, concurrent treatments such as warm compresses, and measurement variability. Because all patients received daily warm compress therapy after treatment, the observed clinical and lipidomic changes should be interpreted as being associated with the overall treatment regimen rather than IPL and TA alone. In addition, only two assessment time points (T0 and T2) were included in this study, and no intermediate or long‐term follow‐up evaluations were performed. Therefore, the temporal progression and durability of treatment effects could not be fully assessed. In addition, the observed high rate of complete resolution may have been influenced by selection bias, the inclusion of relatively mild cases, and the limited follow‐up duration. In addition, no experimental or mechanistic validation was performed in the present study, and therefore, the biological mechanisms underlying the observed lipid alterations remain speculative. Furthermore, information regarding lesion size, lesion location, laterality, and details of prior conservative management was not systematically recorded in the original medical records and therefore could not be included in the analysis. These factors may represent additional sources of heterogeneity and should be considered in future prospective studies.

In addition, lesion size was not quantitatively measured, precluding assessment of the relationship between lesion characteristics and treatment‐associated outcomes. Recurrence beyond the available follow‐up period was not systematically evaluated, and long‐term recurrence rates therefore remain unknown. Adverse events were identified through retrospective review of clinical records rather than a predefined monitoring protocol, which may have resulted in underreporting of minor complications. Outcome assessors were not masked to treatment timing or baseline findings, introducing the potential for observer bias. Furthermore, although all lipid samples were collected by the same investigator using a standardized procedure, the reproducibility of meibum sampling was not formally assessed, and sampling variability may have influenced the lipidomic findings.

In addition, adolescents and children were not included in this study, which may limit the applicability of the findings across different age groups. Therefore, future studies with larger, more diverse populations and prospective controlled designs are needed to validate these findings and further explore the underlying mechanisms.

## 5. Conclusion

This study demonstrated that IPL combined with TA treatment was associated with chalazion resolution and improvements in clinical symptoms and psychological status in this cohort. Significant reductions in meibomian gland lipids, including SMs (Cer and SM) and glycerophospholipids (LPC, LPE, PC, and PE), were observed before and after treatment. These lipidomic alterations may provide preliminary biomarker candidates for future controlled studies and warrant further validation in larger prospective cohorts.

## Author Contributions

Zhenhe Dong and Tao Zhang conceptualized the study. Linxiao Wu, Yongmei Sang, and Huilan Zhang contributed to methodology. Yan Zhang, Tao Zhang, and Yong Tao provided resources. Cheng Zhang and Ran Feng handled visualization. Yan Zhang and Tao Zhang supervised the study. Tao Zhang funded the acquisition.

## Funding

This study was supported by the Beijing Hospitals Authority’s Ascent Program (Grant no. DFL20220301), the Capital Health Development Scientific Research Project Grant (Grant no. 2022‐2‐2035), the Beijing Nova Program (Grant no. 20230484445), and a Grant (Grant no. CX23YQ03) and Excellent Young Talent Innovation Project (Grant no. CX23YQA02) from the Chinese Institutes for Medical Research, Beijing.

## Disclosure

All authors have read and agreed to the published version of the manuscript.

## Conflicts of Interest

The authors declare no conflicts of interest.

## Data Availability

The data that support the findings of this study are available from the corresponding authors upon reasonable request.
